# Crystal structure and Hirshfeld surface analysis of 4-{2,2-di­chloro-1-[(*E*)-(4-fluoro­phen­yl)diazen­yl]ethen­yl}-*N*,*N*-di­methyl­aniline

**DOI:** 10.1107/S2056989020006106

**Published:** 2020-05-06

**Authors:** Kadriye Özkaraca, Mehmet Akkurt, Namiq Q. Shikhaliyev, Ulviyya F. Askerova, Gulnar T. Suleymanova, Irada M. Shikhaliyeva, Ajaya Bhattarai

**Affiliations:** a Institute of Natural and Applied Science, Erciyes University, 38039 Kayseri, Turkey; b Department of Physics, Faculty of Sciences, Erciyes University, 38039 Kayseri, Turkey; c Organic Chemistry Department, Baku State University, Z. Khalilov str. 23, AZ 1148 Baku, Azerbaijan; d Department of Chemistry, M.M.A.M.C (Tribhuvan University), Biratnagar, Nepal

**Keywords:** crystal structure, C—Cl⋯π inter­actions, van der Waals inter­actions, Hirshfeld surface analysis

## Abstract

In the title compound, C_16_H_14_Cl_2_FN_3_, the dihedral angle between the two aromatic rings is 64.12 (14)°. The crystal structure is stabilized by a short Cl⋯H contact, C—Cl⋯π and van der Waals inter­actions. The Hirshfeld surface analysis and two-dimensional fingerprint plots show that H⋯H (33.3%), Cl⋯H/H⋯Cl (22.9%) and C⋯H/H⋯C (15.5%) inter­actions are the most important contributors towards the crystal packing.

## Abstract 2

The dihedral angle between the two aromatic rings of the title compound is 64.12 (14)°. The crystal structure is stabilized by a short Cl⋯H contact, C—Cl⋯π and van der Waals inter­actions.

## Chemical context   

Both inter- and intra­molecular weak inter­actions play a crucial role in determining the properties of organic compounds and controlling their mol­ecular organization in solution and in the solid state, which is sensitive to their chemical environment, solvent polarity, temperature, *etc.* (Asadov *et al.*, 2016 ▸; Maharramov *et al.*, 2009 ▸, 2010 ▸; Mahmudov *et al.*, 2013 ▸, 2014*a* ▸,*b* ▸, 2015 ▸, 2017*a* ▸,*b* ▸, 2019 ▸; Shixaliyev *et al.*, 2013 ▸, 2014 ▸). For example, in catalysis monomeric, oligomeric or polymeric compounds can promote various organic transformations not only by coordination bonds but also through non-covalent inter­actions, such as hydrogen, halogen, chalcogen, pnictogen, tetrel and triel bonds, as well as metal–metal, cation–π, anion–π, lone pair–π, π–π stacking, agostic, pseudo-agostic, anagostic, dispersion-driven, lipophilic, *etc*, or their cooperation (Akbari Afkhami *et al.*, 2017 ▸; Gurbanov *et al.*, 2017 ▸, 2018 ▸; Kopylovich *et al.*, 2011*a* ▸,*b* ▸; Ma *et al.*, 2017*a* ▸,*b* ▸; Mahmoudi *et al.*, 2016 ▸, 2017*a* ▸,*b* ▸,*c* ▸, 2018*a* ▸,*b* ▸). On the other hand, we and other researchers have attached various types of non-covalent-bond donor synthons to dye mol­ecules, which results in inter­esting analytical and solvatochromic properties (Maharramov *et al.*, 2018 ▸; Mahmudov *et al.*, 2010 ▸, 2011 ▸; Mahmudov & Pombeiro, 2016 ▸).
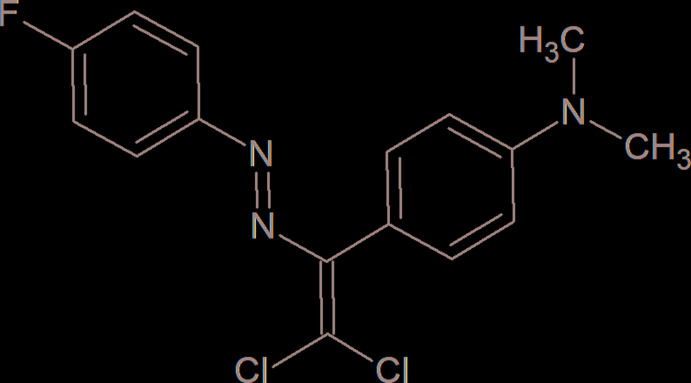


In order to continue our work in this direction, we have functionalized a new azo dye, 4-{2,2-di­chloro-1-[(*E*)-(4-fluoro­phen­yl)diazen­yl]ethen­yl}-*N*,*N*-di­methyl­aniline, which provides C—H⋯F and C—Cl⋯F types of inter­molecular weak inter­actions.

## Structural commentary   

**Figure 1:**
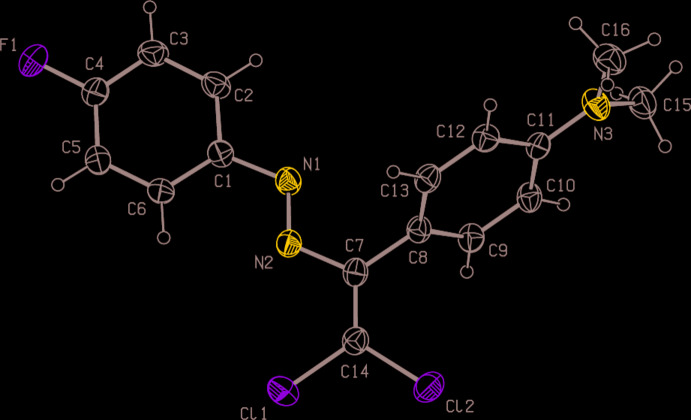
The mol­ecular structure of the title compound, showing the atom labelling and displacement ellipsoids drawn at the 30% probability level.

In the title compound (Fig. 1 ▸), the dihedral angle between the benzene rings (C1–C6 and C8–C13) of the 4-fluoro­phenyl and *N*,*N*-di­methyl­aniline groups is 64.12 (14)°. The amine N atom as well as the directly adjacent arene C atom are bent a little out of the plane of the other five aromatic C atoms, with deviations of 0.007 (3) Å for C11 and 0.027 (2) Å for N3. The N1—N2—C7—C14, N2—C7—C14—Cl1, N2—C7—C14—Cl2 and C8—C7—C14—Cl2 torsion angles are −172.0 (2), 2.1 (3), −177.0 (2) and 0.6 (4)°, respectively.

## Supra­molecular features and Hirshfeld surface analysis   

**Figure 2:**
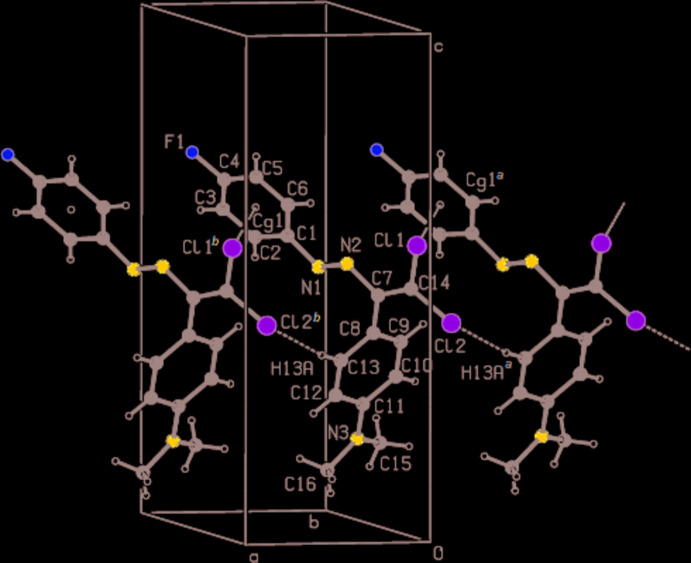
A partial packing diagram of the title compound showing chain formation along the *a-*axis direction. Symmetry operators: (*a*) −1 + *x*, *y*, *z*; (*b*) 1 + *x*, *y*, *z. *Cg**1 is the centroid of the C1–C6 ring.

In the crystal, the mol­ecules are connected by a short Cl2⋯H13*A* contact (2.96 Å) and C—Cl⋯π inter­actions, which contribute to the overall packing energy stabilization, into infinite columns along the *a*-axis direction (Table 1 ▸; Fig. 2 ▸).

**Figure 3:**
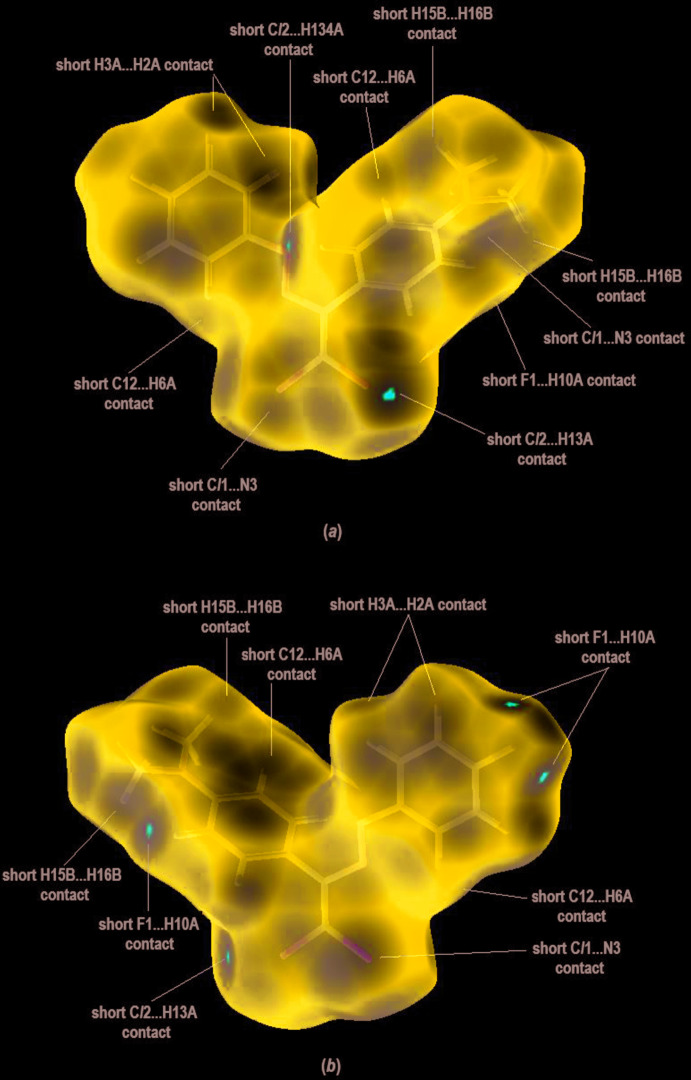
Front and back sides of the three-dimensional Hirshfeld surface of the title compound plotted over *d*_norm_ in the range 0.0350 to 0.8404 a.u.

In order to visualize the inter­molecular inter­actions in the crystal of the title compound, a Hirshfeld surface analysis (Hirshfeld, 1977 ▸; Spackman & Jayatilaka, 2009 ▸) was carried out using *CrystalExplorer17.5* (Turner *et al.*, 2017 ▸). Three-dimensional mol­ecular Hirshfeld surfaces were generated using a ‘high standard’ surface resolution colour-mapped over the normalized contact distance. The red, white and blue regions visible on the *d*_norm_ surfaces indicate contacts with distances shorter, longer and equal to the van der Waals radii (Fig. 3 ▸).

**Figure 4:**
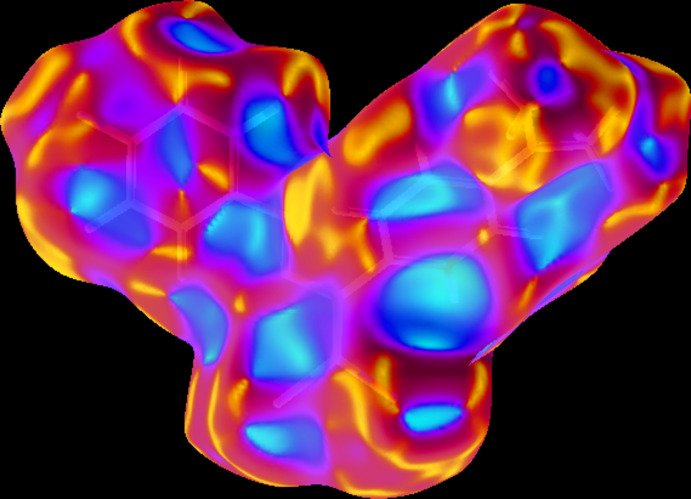
Hirshfeld surface of the title compound plotted over shape-index.

The bright-red spots near atoms Cl2 and C13 in Fig.3a refer to the short Cl2⋯H13*A* contact, and near the atoms F1 and C10 in Fig. 3 ▸ *b* to the F1⋯H10*A* contact. The shape-index of the Hirshfeld surface is a tool to visualize the π–π stacking by the presence of adjacent red and blue triangles; if there are no adjacent red and/or blue triangles, then there are no π–π inter­actions. Fig. 4 ▸ clearly suggests that there are no π–π inter­actions in the crystal structure.

**Figure 5:**
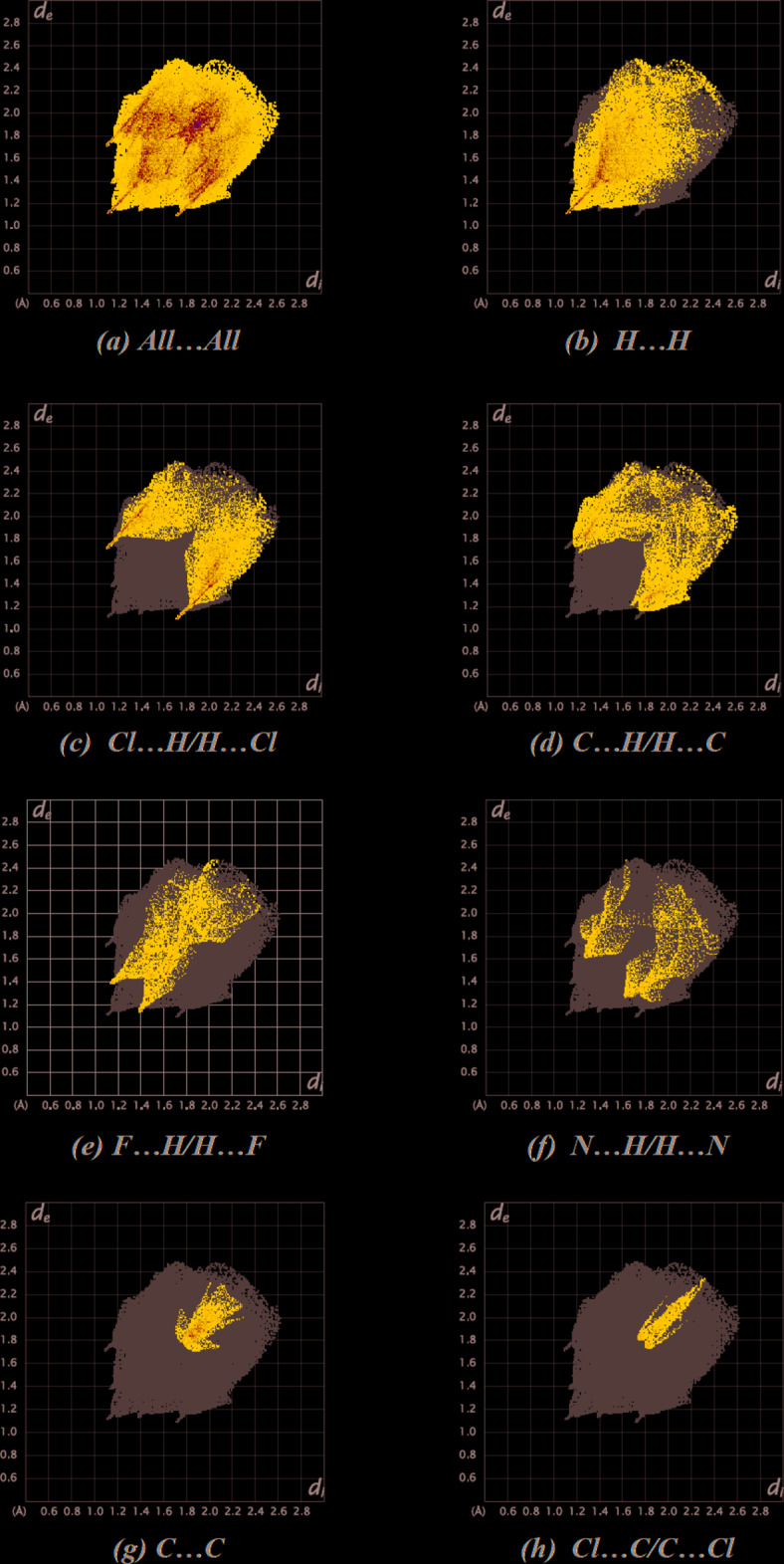
The full two-dimensional fingerprint plots for the title compound, showing (*a*) all inter­actions, and delineated into (*b*) H⋯H, (*c*) Cl⋯H/H⋯Cl, (*d*) C⋯H/H⋯C, (*e*) F⋯H/H⋯F, (*f*) N⋯H/H⋯N, (*g*) C⋯C and (*h*) Cl⋯C/C⋯Cl inter­actions. The *d*_i_ and *d*_e_ values are the closest inter­nal and external distances (in Å) from given points on the Hirshfeld surface.

The overall two-dimensional fingerprint plot, Fig. 5 ▸ *a*, and those delineated into H⋯H, Cl⋯H/H⋯Cl, C⋯H/H⋯C, F⋯H/H⋯F, N⋯H/H⋯N, C⋯C and Cl⋯C/C⋯Cl contacts (McKinnon *et al.*, 2007 ▸) are illustrated in Fig. 5 ▸ *b*–*h*, together with their relative contributions to the Hirshfeld surface while details of the various contacts are given in Table 2 ▸. The most important inter­action is H⋯H, contributing 33.3% to the overall crystal packing, which is reflected in Fig. 5 ▸ *b* as widely scattered points of high density due to the large hydrogen content of the mol­ecule with the tip at *d*_e_ = *d*_i_ = 1.10 Å. The reciprocal Cl⋯H/H⋯Cl inter­actions appear as two sym­metrical broad wings with *d*_e_ + *d*_i_ ≃ 2.80 Å and contribute 22.9% to the Hirshfeld surface (Fig. 5 ▸ *c*). The pair of characteristic wings in the fingerprint plot delineated into H⋯C/C⋯H contacts (Fig. 5 ▸ *d*; 15.5% contribution to the Hirshfeld surface), have the tips at *d*_e_ + *d*_i_ ≃ 2.95 Å. The fingerprint plot for F⋯H/H⋯F contacts (9.0% contribution), Fig. 5 ▸ *e*, has a pair of spikes with the tips at *d*_e_ + *d*_i_ = 2.55 Å. The remaining contributions from the other different inter­atomic contacts to the Hirshfeld surfaces are listed in Table 3 ▸. The small contribution of the other weak inter­molecular N⋯H/H⋯N, C⋯C, Cl⋯C/C⋯Cl, N⋯C/C⋯N, Cl⋯N/N⋯Cl, Cl⋯F/F⋯Cl, C⋯F/F⋯C and F⋯N/N⋯F contacts has a negligible effect on the packing.

The Hirshfeld surface analysis confirms the importance of H-atom contacts in establishing the packing. The large number of H⋯H, Cl⋯H/H⋯Cl and C⋯H/H⋯C inter­actions suggest that van der Waals inter­actions and hydrogen bonding play the major roles in the crystal packing (Hathwar *et al.*, 2015 ▸).

## Database survey   

A search of the Cambridge Structural Database (CSD, Version 5.41, update of November 2019; Groom *et al.*, 2016 ▸) for structures having an (*E*)-1-(2,2-di­chloro-1-phenyl­vin­yl)-2-phenyl­diazene unit gave 25 hits. Six compounds closely resemble the title compound, *viz*. 1-(4-bromo­phen­yl)-2-[2,2-di­chloro-1-(4-nitro­phen­yl)ethen­yl]diazene (CSD refcode HONBOE; Akkurt *et al.*, 2019 ▸), 1-(4-chloro­phen­yl)-2-[2,2-di­chloro-1-(4-nitro­phen­yl)ethen­yl]diazene (HONBUK; Akkurt *et al.*, 2019 ▸), 1-(4-chloro­phen­yl)-2-[2,2-di­chloro-1-(4-fluoro­phen­yl)ethen­yl]diazene (HODQAV; Shikhaliyev *et al.*, 2019 ▸), 1-[2,2-di­chloro-1-(4-nitro­phen­yl)ethen­yl]-2-(4-fluoro­phen­yl)diazene (XIZREG; Atioğlu *et al.*, 2019 ▸), 1,1-[methyl­ene­bis(4,1-phenyl­ene)]bis­[(2,2-di­chloro-1-(4-nitro­phen­yl)ethen­yl]diazene (LEQXIR; Shixaliyev *et al.*, 2018 ▸), 1,1-[methyl­enebis(4,1-phenyl­ene)]bis­{[2,2-di­chloro-1-(4-chloro­phen­yl) ethen­yl]diazene} (LEQXOX; Shikhaliyev *et al.*, 2018 ▸).

In the crystal structures of HONBOE and HONBUK, the aromatic rings form dihedral angles of 60.9 (2) and 64.1 (2)°, respectively. Mol­ecules are linked through weak *X*⋯Cl contacts (*X* = Br for HONBOE and Cl for HONBUK) and C—H⋯Cl and C—Cl⋯π inter­actions into sheets parallel to the *ab* plane. Additional van der Waals inter­actions consolidate the three-dimensional packing. In the crystal of HODQAV, mol­ecules are stacked in columns along the *a* axis *via* weak C—H⋯Cl hydrogen bonds and face-to-face π–π stacking inter­actions. The crystal packing is further stabilized by short Cl⋯Cl contacts. In XIZREG, mol­ecules are linked by C—H⋯O hydrogen bonds into zigzag chains running along the *c*-axis direction. The crystal packing is further stabilized by C—Cl⋯π, C—F⋯π and N—O⋯π inter­actions. In the crystal of LEQXIR, C—H⋯N and C—H⋯O hydrogen bonds and Cl⋯O contacts were found, and in LEQXOX, C—H⋯N and Cl⋯Cl contacts are observed.

## Synthesis and crystallization   

The title compound was synthesized according to the reported method (Shixaliyev *et al.*, 2018 ▸). A 20 mL screw-neck vial was charged with DMSO (10mL), (*E*)-4-{[2-(4-fluoro­phen­yl)hydrazono]meth­yl}-*N*,*N*-di­methyl­aniline (257 mg, 1 mmol), tetra­methyl­ethylenedi­amine (TMEDA) (295 mg, 2.5 mmol), CuCl (2 mg, 0.02 mmol) and CCl_4_ (20 mmol, 10 equiv). After 1–3 h (until TLC analysis showed complete consumption of the corresponding Schiff base), the reaction mixture was poured into ∼0.01 *M* solution of HCl (100 mL, pH = 2–3), and extracted with di­chloro­methane (3 × 20 mL). The combined organic phase was washed with water (3 × 50 mL), brine (30 mL), dried over anhydrous Na_2_SO_4_ and concentrated *in vacuo* using a rotary evaporator. The residue was purified by column chromatography on silica gel using appropriate mixtures of hexane and di­chloro­methane (3/1–1/1). Crystals suitable for X-ray analysis were obtained by slow evaporation of an ethanol solution. Orange solid (78%); m.p. 418 K. Analysis calculated for C_16_H_14_Cl_2_FN_3_ (*M* = 338.21): C 56.82, H 4.17, N 12.42; found: C 56.78, H 4.11, N 12.34%. ^1^H NMR (300 MHz, CDCl_3_) δ (ppm) 3.05 (6H, NMe_2_), 6.79–7.86 (8H, Ar). ^13^C NMR (75 MHz, CDCl_3_) δ (ppm) 134.71, 131.08, 130.42, 128.97, 128.85, 125.34, 125.22, 124.68, 124.57, 119.46, 116.13, 115.83, 115.47, 115.17, 115.12, 111.49, 110.83, 43.94, 40.31. ESI–MS: *m*/*z*: 338.12 [*M*+H]^+^.

## Refinement   

Crystal data, data collection and structure refinement details are summarized in Table 4 ▸. All C-bound H atoms were refined using a riding model with *d*(C—H) = 0.93 Å, *U*_iso_(H) = 1.2*U*_eq_(C) for aromatic and 0.96 Å, *U*_iso_(H) = 1.5*U*_eq_(C) for methyl H atoms.

## Supplementary Material

Crystal structure: contains datablock(s) I. DOI: 10.1107/S2056989020006106/vm2232sup1.cif

Structure factors: contains datablock(s) I. DOI: 10.1107/S2056989020006106/vm2232Isup2.hkl

Supporting information file. DOI: 10.1107/S2056989020006106/vm2232Isup3.cml

CCDC reference: 2001173

Additional supporting information:  crystallographic information; 3D view; checkCIF report

## Figures and Tables

**Table 1:** C—Cl⋯π interaction geometry (Å, °) *Cg*1 is the centroid of the C1–C6 ring.

*D*—H⋯*A*	*D*—H	H⋯*A*	*D*⋯*A*	*D*—H⋯*A*
C14—Cl1⋯*Cg*1^i^	1.72 (1)	3.93 (1)	3.882 (3)	76 (1)

Symmetry code: (i) 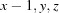.

**Table 2:** Summary of short inter­atomic contacts (Å) in the title compound

*A*⋯*B*	Distance	Symmetry operation for *B*
H15*B*⋯H16*B*	2.45	−1 + *x*, *y*, *z*
Cl1⋯N3	3.409 (3)	− 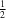 − *x*, 1 − *y*, 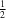 + *z*
C12⋯H6*A*	2.97	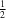 − *x*, 1 − *y*, − 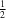 + *z*
Cl2⋯H13*A*	2.96	−1 + *x*, *y*, *z*
F1⋯H10*A*	2.66	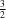 + *x*, 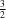 − *y*, 1 − *z*
H3*A*⋯H2*A*	2.53	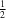 + *x*, 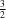 − *y*, 1 − *z*

**Table 3:** Percentage contributions of inter­atomic contacts to the Hirshfeld surface for the title compound

Contact	Percentage contribution
H⋯H	33.3
Cl⋯H/H⋯Cl	22.9
C⋯H/H⋯C	15.5
F⋯H/H⋯F	9.0
N⋯H/H⋯N	4.9
C⋯C	4.7
Cl⋯C/C⋯Cl	2.7
N⋯C/C⋯N	2.0
Cl⋯N/N⋯Cl	2.0
Cl⋯F/F⋯Cl	1.9
C⋯F/F⋯C	0.8
F⋯N/N⋯F	0.3

**Table 4:** Experimental details

Crystal data	
Chemical formula	C_16_H_14_Cl_2_FN_3_
*M*_r_	338.20
Crystal system, space group	Orthorhombic, *P*2_1_2_1_2_1_
Temperature (K)	296
*a*, *b*, *c* (Å)	6.0730 (3), 15.9782 (9), 16.3860 (7)
*V* (Å^3^)	1590.03 (14)
*Z*	4
Radiation type	Mo *K*α
μ (mm^−1^)	0.42
Crystal size (mm)	0.27 × 0.24 × 0.17

Data collection	
Diffractometer	Bruker APEXII PHOTON 100 detector
Absorption correction	Multi-scan (*SADABS*; Bruker, 2003 ▸)
*T*_min_, *T*_max_	0.887, 0.944
No. of measured, independent and observed [*I* > 2σ(*I*)] reflections	12082, 3215, 2696
*R*_int_	0.036
(sin θ/λ)_max_ (Å^−1^)	0.625

Refinement	
*R*[*F* ^2^ > 2σ(*F* ^2^)], *wR*(*F* ^2^), *S*	0.034, 0.084, 1.05
No. of reflections	3215
No. of parameters	201
H-atom treatment	H-atom parameters constrained
Δρ_max_, Δρ_min_ (e Å^−3^)	0.13, −0.22
Absolute structure	Flack *x* determined using 1002 quotients [(*I* ^+^)−(*I* ^−^)]/[(*I* ^+^)+(*I* ^−^)] (Parsons *et al.*, 2013 ▸).
Absolute structure parameter	−0.02 (2)

Computer programs: *APEX3* and *SAINT* (Bruker, 2007 ▸), *SHELXT2016/6* (Sheldrick, 2015*a* ▸), *SHELXL2016/6* (Sheldrick, 2015*b* ▸), *ORTEP-3 for Windows* (Farrugia, 2012 ▸) and *PLATON* (Spek, 2020 ▸).

## supplementary crystallographic information

### Crystal data

**Table:**

C_16_H_14_Cl_2_FN_3_	*D*_x_ = 1.413 Mg m^−^^3^
*M**_r_* = 338.20	Mo *K*α radiation, λ = 0.71073 Å
Orthorhombic, *P*2_1_2_1_2_1_	Cell parameters from 6056 reflections
*a* = 6.0730 (3) Å	θ = 2.8–26.4°
*b* = 15.9782 (9) Å	µ = 0.42 mm^−^^1^
*c* = 16.3860 (7) Å	*T* = 296 K
*V* = 1590.03 (14) Å^3^	Block, orange
*Z* = 4	0.27 × 0.24 × 0.17 mm
*F*(000) = 696	

### Data collection

**Table:**

Bruker APEXII PHOTON 100 detector diffractometer	2696 reflections with *I* > 2σ(*I*)
φ and ω scans	*R*_int_ = 0.036
Absorption correction: multi-scan (SADABS; Bruker, 2003)	θ_max_ = 26.4°, θ_min_ = 2.5°
*T*_min_ = 0.887, *T*_max_ = 0.944	*h* = −7→7
12082 measured reflections	*k* = −19→19
3215 independent reflections	*l* = −20→20

### Refinement

**Table:**

Refinement on *F*^2^	Hydrogen site location: inferred from neighbouring sites
Least-squares matrix: full	H-atom parameters constrained
*R*[*F*^2^ > 2σ(*F*^2^)] = 0.034	*w* = 1/[σ^2^(*F*_o_^2^) + (0.0431*P*)^2^ + 0.1514*P*] where *P* = (*F*_o_^2^ + 2*F*_c_^2^)/3
*wR*(*F*^2^) = 0.084	(Δ/σ)_max_ < 0.001
*S* = 1.05	Δρ_max_ = 0.13 e Å^−^^3^
3215 reflections	Δρ_min_ = −0.22 e Å^−^^3^
201 parameters	Absolute structure: Flack *x* determined using 1002 quotients [(*I*^+^)-(*I*^-^)]/[(*I*^+^)+(*I*^-^)] (Parsons *et al.*, 2013).
0 restraints	Absolute structure parameter: −0.02 (2)

### Special details

**Table:**

Geometry. All esds (except the esd in the dihedral angle between two l.s. planes) are estimated using the full covariance matrix. The cell esds are taken into account individually in the estimation of esds in distances, angles and torsion angles; correlations between esds in cell parameters are only used when they are defined by crystal symmetry. An approximate (isotropic) treatment of cell esds is used for estimating esds involving l.s. planes.

### Fractional atomic coordinates and isotropic or equivalent isotropic displacement parameters (Å^2^)

**Table:**

	*x*	*y*	*z*	*U*_iso_*/*U*_eq_	
C1	0.4252 (4)	0.61833 (16)	0.56623 (16)	0.0443 (6)	
C2	0.5620 (5)	0.68715 (18)	0.55987 (18)	0.0509 (7)	
H2A	0.540332	0.725536	0.517976	0.061*	
C3	0.7298 (5)	0.6994 (2)	0.61488 (17)	0.0573 (8)	
H3A	0.823241	0.745307	0.610610	0.069*	
C4	0.7553 (5)	0.6416 (2)	0.67653 (18)	0.0594 (8)	
C5	0.6205 (6)	0.5739 (2)	0.6855 (2)	0.0673 (9)	
H5A	0.641740	0.536178	0.728082	0.081*	
C6	0.4535 (5)	0.56267 (19)	0.63053 (18)	0.0574 (8)	
H6A	0.358152	0.517491	0.636245	0.069*	
C7	−0.0179 (4)	0.53364 (16)	0.45205 (16)	0.0439 (6)	
C8	−0.0207 (4)	0.58320 (16)	0.37493 (15)	0.0421 (6)	
C9	−0.1979 (4)	0.63333 (18)	0.35356 (16)	0.0465 (6)	
H9A	−0.319781	0.635689	0.387856	0.056*	
C10	−0.1976 (4)	0.67976 (18)	0.28261 (16)	0.0470 (7)	
H10A	−0.319254	0.712725	0.270104	0.056*	
C11	−0.0185 (4)	0.67833 (17)	0.22914 (15)	0.0419 (6)	
C12	0.1597 (5)	0.62650 (17)	0.25034 (16)	0.0463 (6)	
H12A	0.280540	0.622891	0.215669	0.056*	
C13	0.1577 (5)	0.58098 (17)	0.32175 (17)	0.0473 (6)	
H13A	0.278742	0.547916	0.334730	0.057*	
C14	−0.1613 (5)	0.47174 (16)	0.46824 (16)	0.0469 (6)	
C15	−0.1744 (6)	0.7909 (2)	0.14541 (19)	0.0624 (8)	
H15A	−0.174793	0.828007	0.191458	0.094*	
H15B	−0.318008	0.766689	0.138916	0.094*	
H15C	−0.136230	0.821563	0.097069	0.094*	
C16	0.1532 (6)	0.7139 (2)	0.09817 (19)	0.0666 (8)	
H16A	0.162051	0.655738	0.083877	0.100*	
H16B	0.292048	0.732038	0.119880	0.100*	
H16C	0.118356	0.746158	0.050458	0.100*	
Cl1	−0.16032 (15)	0.41361 (5)	0.55620 (5)	0.0677 (3)	
Cl2	−0.36345 (14)	0.44180 (5)	0.40139 (5)	0.0680 (3)	
F1	0.9220 (4)	0.65309 (16)	0.73030 (13)	0.0957 (8)	
N1	0.2619 (4)	0.60931 (14)	0.50431 (14)	0.0477 (5)	
N2	0.1433 (4)	0.54576 (14)	0.51363 (13)	0.0469 (5)	
N3	−0.0159 (4)	0.72557 (16)	0.15854 (14)	0.0554 (6)	

### Atomic displacement parameters (Å^2^)

**Table:**

	*U*^11^	*U*^22^	*U*^33^	*U*^12^	*U*^13^	*U*^23^
C1	0.0531 (15)	0.0378 (14)	0.0420 (13)	0.0006 (11)	−0.0011 (12)	−0.0018 (11)
C2	0.0671 (18)	0.0421 (15)	0.0436 (14)	−0.0075 (13)	0.0032 (14)	0.0000 (12)
C3	0.0676 (19)	0.0562 (17)	0.0480 (16)	−0.0201 (15)	0.0029 (15)	−0.0036 (14)
C4	0.0617 (18)	0.070 (2)	0.0468 (15)	−0.0164 (16)	−0.0090 (15)	0.0004 (15)
C5	0.079 (2)	0.065 (2)	0.0583 (18)	−0.0209 (17)	−0.0234 (17)	0.0185 (15)
C6	0.0655 (18)	0.0480 (17)	0.0588 (17)	−0.0192 (15)	−0.0148 (15)	0.0152 (14)
C7	0.0430 (13)	0.0427 (14)	0.0461 (14)	0.0082 (11)	−0.0043 (12)	−0.0006 (11)
C8	0.0433 (13)	0.0398 (14)	0.0433 (13)	0.0034 (11)	−0.0052 (12)	−0.0006 (11)
C9	0.0409 (14)	0.0550 (16)	0.0436 (14)	0.0078 (12)	0.0017 (12)	−0.0020 (12)
C10	0.0421 (14)	0.0513 (15)	0.0474 (15)	0.0121 (12)	−0.0034 (13)	0.0028 (12)
C11	0.0416 (14)	0.0435 (15)	0.0406 (14)	0.0010 (11)	−0.0047 (12)	−0.0043 (11)
C12	0.0384 (13)	0.0517 (15)	0.0486 (14)	0.0059 (12)	0.0035 (13)	−0.0033 (12)
C13	0.0392 (13)	0.0478 (14)	0.0547 (15)	0.0077 (12)	−0.0061 (13)	−0.0012 (12)
C14	0.0453 (14)	0.0438 (14)	0.0515 (14)	0.0037 (12)	−0.0048 (13)	−0.0001 (11)
C15	0.0624 (19)	0.0655 (19)	0.0591 (18)	0.0090 (16)	−0.0018 (17)	0.0153 (15)
C16	0.0647 (19)	0.075 (2)	0.0595 (18)	0.0020 (18)	0.0144 (18)	0.0095 (16)
Cl1	0.0720 (5)	0.0627 (5)	0.0683 (5)	−0.0060 (4)	0.0004 (5)	0.0197 (4)
Cl2	0.0576 (4)	0.0620 (5)	0.0845 (5)	−0.0089 (4)	−0.0209 (4)	0.0044 (4)
F1	0.0914 (16)	0.1181 (18)	0.0777 (13)	−0.0488 (14)	−0.0399 (12)	0.0172 (13)
N1	0.0566 (14)	0.0415 (12)	0.0450 (12)	−0.0009 (11)	−0.0058 (11)	0.0018 (10)
N2	0.0506 (12)	0.0449 (12)	0.0454 (11)	−0.0031 (11)	−0.0081 (11)	0.0016 (10)
N3	0.0568 (15)	0.0634 (16)	0.0461 (12)	0.0129 (12)	0.0076 (12)	0.0089 (11)

### Geometric parameters (Å, º)

**Table:**

C1—C2	1.382 (4)	C10—C11	1.397 (4)
C1—C6	1.389 (4)	C10—H10A	0.9300
C1—N1	1.426 (3)	C11—N3	1.381 (3)
C2—C3	1.374 (4)	C11—C12	1.406 (4)
C2—H2A	0.9300	C12—C13	1.378 (4)
C3—C4	1.377 (4)	C12—H12A	0.9300
C3—H3A	0.9300	C13—H13A	0.9300
C4—F1	1.354 (3)	C14—Cl2	1.713 (3)
C4—C5	1.365 (4)	C14—Cl1	1.715 (3)
C5—C6	1.369 (4)	C15—N3	1.436 (4)
C5—H5A	0.9300	C15—H15A	0.9600
C6—H6A	0.9300	C15—H15B	0.9600
C7—C14	1.344 (4)	C15—H15C	0.9600
C7—N2	1.419 (3)	C16—N3	1.438 (4)
C7—C8	1.491 (4)	C16—H16A	0.9600
C8—C9	1.387 (4)	C16—H16B	0.9600
C8—C13	1.391 (4)	C16—H16C	0.9600
C9—C10	1.379 (4)	N1—N2	1.255 (3)
C9—H9A	0.9300		
			
C2—C1—C6	119.5 (3)	N3—C11—C10	121.7 (2)
C2—C1—N1	116.4 (2)	N3—C11—C12	121.3 (2)
C6—C1—N1	124.1 (2)	C10—C11—C12	117.0 (2)
C3—C2—C1	120.6 (3)	C13—C12—C11	120.9 (3)
C3—C2—H2A	119.7	C13—C12—H12A	119.5
C1—C2—H2A	119.7	C11—C12—H12A	119.5
C2—C3—C4	118.0 (3)	C12—C13—C8	121.7 (2)
C2—C3—H3A	121.0	C12—C13—H13A	119.1
C4—C3—H3A	121.0	C8—C13—H13A	119.1
F1—C4—C5	119.0 (3)	C7—C14—Cl2	122.9 (2)
F1—C4—C3	118.1 (3)	C7—C14—Cl1	124.2 (2)
C5—C4—C3	122.9 (3)	Cl2—C14—Cl1	112.87 (16)
C4—C5—C6	118.5 (3)	N3—C15—H15A	109.5
C4—C5—H5A	120.8	N3—C15—H15B	109.5
C6—C5—H5A	120.8	H15A—C15—H15B	109.5
C5—C6—C1	120.5 (3)	N3—C15—H15C	109.5
C5—C6—H6A	119.8	H15A—C15—H15C	109.5
C1—C6—H6A	119.8	H15B—C15—H15C	109.5
C14—C7—N2	114.0 (2)	N3—C16—H16A	109.5
C14—C7—C8	123.4 (2)	N3—C16—H16B	109.5
N2—C7—C8	122.5 (2)	H16A—C16—H16B	109.5
C9—C8—C13	117.5 (2)	N3—C16—H16C	109.5
C9—C8—C7	122.0 (2)	H16A—C16—H16C	109.5
C13—C8—C7	120.6 (2)	H16B—C16—H16C	109.5
C10—C9—C8	121.5 (3)	N2—N1—C1	113.2 (2)
C10—C9—H9A	119.2	N1—N2—C7	114.8 (2)
C8—C9—H9A	119.2	C11—N3—C15	121.0 (2)
C9—C10—C11	121.4 (2)	C11—N3—C16	120.9 (2)
C9—C10—H10A	119.3	C15—N3—C16	118.0 (2)
C11—C10—H10A	119.3		
			
C6—C1—C2—C3	2.2 (4)	N3—C11—C12—C13	178.6 (3)
N1—C1—C2—C3	−177.5 (2)	C10—C11—C12—C13	−1.4 (4)
C1—C2—C3—C4	−0.6 (4)	C11—C12—C13—C8	1.1 (4)
C2—C3—C4—F1	179.3 (3)	C9—C8—C13—C12	−0.1 (4)
C2—C3—C4—C5	−0.7 (5)	C7—C8—C13—C12	−179.7 (3)
F1—C4—C5—C6	−179.6 (3)	N2—C7—C14—Cl2	−177.0 (2)
C3—C4—C5—C6	0.4 (6)	C8—C7—C14—Cl2	0.6 (4)
C4—C5—C6—C1	1.2 (5)	N2—C7—C14—Cl1	2.1 (3)
C2—C1—C6—C5	−2.5 (5)	C8—C7—C14—Cl1	179.6 (2)
N1—C1—C6—C5	177.1 (3)	C2—C1—N1—N2	−179.9 (2)
C14—C7—C8—C9	63.2 (4)	C6—C1—N1—N2	0.4 (4)
N2—C7—C8—C9	−119.4 (3)	C1—N1—N2—C7	−178.6 (2)
C14—C7—C8—C13	−117.3 (3)	C14—C7—N2—N1	−172.0 (2)
N2—C7—C8—C13	60.1 (3)	C8—C7—N2—N1	10.4 (3)
C13—C8—C9—C10	−0.4 (4)	C10—C11—N3—C15	13.1 (4)
C7—C8—C9—C10	179.1 (3)	C12—C11—N3—C15	−166.9 (3)
C8—C9—C10—C11	0.0 (4)	C10—C11—N3—C16	−170.4 (3)
C9—C10—C11—N3	−179.1 (3)	C12—C11—N3—C16	9.6 (4)
C9—C10—C11—C12	0.9 (4)		

### Hydrogen-bond geometry (Å, º)

*Cg*1 is the centroid of the C1–C6 ring.

**Table:**

*D*—H···*A*	*D*—H	H···*A*	*D*···*A*	*D*—H···*A*
C14—Cl1···*Cg*1^i^	1.72 (1)	3.93 (1)	3.882 (3)	76 (1)

Symmetry code: (i) *x*−1, *y*, *z*.
